# Peptide-Functionalized Gold Nanorods as a Model to Reach the Cell Nucleus: Synthesis and Structural Characterizations in View of Theragnostic Applications

**DOI:** 10.1021/acs.jpcb.5c07563

**Published:** 2026-03-16

**Authors:** Ludovica Binelli, Federica Bertelà, Simone Amatori, Diego Lipani, Chiara Battocchio, Giovanna Iucci, Luca Tortora, Valentina Dini, Sveva Grande, Alessandra Palma, Marco Ranaldi, Barbara De Berardis, Maria G. Ammendolia, Carlo Mancini-Terraciano, Andrea Fabbri, Andrea Attili, Teresa Scotognella, Alessandro Giordano, Maria L. Calcagni, Monica Dettin, Annj Zamuner, Valentin-Adrian Maraloiu, Iole Venditti

**Affiliations:** † Sciences Department, 19012 Roma Tre University, 00146 Rome, Italy; ‡ Istituto Nazionale di Fisica Nucleare (INFN), Sezione di Roma3, Department of Sciences, Roma Tre University, 00146 Rome, Italy; § CERIC-ERIC, S. S. 14 - km 163,5 in AREA Science Park, Basovizza, Trieste 34149, Italy; ∥ National Center for Innovative Technologies in Public Health, 9289 Istituto Superiore di Sanità, 00161 Rome, Italy; ⊥ Istituto Nazionale di Fisica Nucleare (INFN), Sezione di Roma1, Department of Physics, University La Sapienza, 00185 Rome, Italy; # Nuclear Medicine Unit, Fondazione Policlinico Universitario A. Gemelli IRCCS, 00168 Rome, Italy; ¶ Nuclear Medicine Institute, Università Cattolica del Sacro Cuore, 00168 Rome, Italy; ∇ Department of Industrial Engineering, University of Padova, 35131 Padova, Italy; ○ National Institute of Materials Physics, 077125 Magurele, Romania

## Abstract

Gold nanoparticles are proving to be highly successful for delivering drugs to specific targets, exploiting carefully designed functionalizations. This work creates and optimizes the synthesis of gold nanorods (AuNRs), subsequently functionalized with a peptide, TAT, appropriately modified to allow attachment to the rods and guide their entry into the cell nucleus and nuclear growth. Various chemical and physical characterizations were performed to verify and optimize the AuNRs-TAT system. DLS, Z-potential, UV–vis, and FT-IR spectroscopies confirmed the nanosize, monodispersity, colloidal stability, and successful functionalization. Furthermore, structural characterizations conducted using synchrotron radiation were crucial for understanding the actual interaction between the gold surface and the modified TAT peptide. The study highlighted how this material is indeed a good drug delivery system, stable over time, and promising for reaching the cell nucleus.

## Graphical abstract

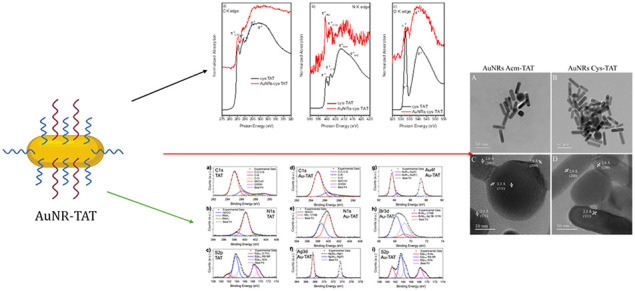

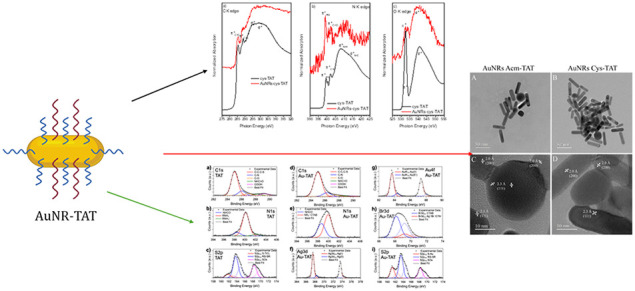

## 1 Introduction

The growing need to create increasingly efficient drugs capable of performing multiple functions simultaneously has led to the development of theragnostic systems and personalized medicine.^1,2^ In these fields, nanomaterials have proven to be an amazing tool, which in the past decade have been widely used in various advanced applications, from energy to optics to drug delivery.^3–8^ Many radiopharmaceuticals used in nuclear medicine can also potentially form the basis for the development of theranostic systems when conjugated on nanoparticles such as appropriately functionalized gold nanorods (AuNRs). These nanostructures possess a special optical property, the localized surface plasmon resonance (LSPR), which can be easily visualized using UV–visible spectroscopy, obtaining a spectrum with two distinct absorption peaks, the first linked to the longitudinal side (wavelength approximately 700 nm) and the second to the transverse side (wavelength approximately 510 nm).^9,10^ Another advantage that AuNRs possess is a high surface-to-volume ratio that allows a high degree of functionalization, even with multiple molecules simultaneously.^11^ As is known, nanomaterials can modify their properties by modifying their surface for greater solubility in water or their shape and size for greater biocompatibility and bioavailability. But one of the major problems in the use of nanomaterials, and in particular AuNRs, is how they can penetrate cells and, more specifically, the nucleus. Literature studies have shown that CTAB-surfaced AuNRs are able to enter cells by nonreceptor-mediated endocytosis. The vesicles that transport AuNRs show no abnormalities in either shape or membrane composition and are located near the cell membrane.^12^ So, the real big hurdle would seem to be bringing the AuNRs into the nucleus. To resolve this issue, recent studies have demonstrated that cell-penetrating peptides (CPPs) can permit the transduction of molecules or nanoparticles. The first peptide of the CPP class was identified in the late 1980s with the discovery of the TAT peptide, encoded by the human immunodeficiency virus type 1 (HIV-1), by Frankel and Pabo,^13^ who demonstrated that the TAT peptide could enter cells and translocate into the nucleus. CPPs present a breakthrough as delivery systems for macromolecules. CPPs are capable of entering the body in a noninvasive manner, do not destroy the integrity of cellular membranes, and are considered to be highly efficient and safe. Thus far, CPPs have been used to safely deliver peptides, proteins, and nucleic acids that are generally difficult to deliver due to some of their inherent properties. The CPPs have been used with a wide variety of cell types and in combination with different cargos. However, the exact mechanism that CPPs use to cross cell membranes is still an unsolved issue. It is well-known that CPPs use different mechanisms to enter cells. In general, the mechanisms can be associated with two broad groups: endocytosis and direct penetration. In the literature, it is described that many CPPs use different uptake pathways depending on their structure, net charge, concentration, type of cargo, cell lines used, temperature at which uptake studies were conducted, and incubation times.^14,15^ Basically, the TAT’s domain encompasses amino acids 38–58 (the basic region of TAT), maintaining the property of transduction enabling both its nuclear and cytoplasmic accumulation.^16^ The basic region of TAT, like that of the HIV-1 original protein, did not have groups that could permit the loading on a gold surface. As is well-known, there are many ways to functionalize the AuNR surface. One of the most fruitful functionalization methods is the direct thiol reaction, which allows stable binding between the molecule and the nanorods.^17^ Santos-Cuevas et al. have provided the solution of how to label the TAT on the surface by modifying the carboxyl terminal and adding a free cysteine. In this case, it is the thiol group that permits the loading.^18^

This work optimizes the synthesis of gold nanorods and their functionalization with a suitably modified TAT peptide to allow for cell nucleus growth. Various chemical and physical characterizations were performed to verify and optimize the system, such as DLS and Z-potential measurements and UV–vis and FT-IR spectroscopies. Furthermore, the structural characterizations conducted using synchrotron radiation were crucial and confirmed the amazing perspective of this system in nanomedicine.

## 2 Materials and Methods

### 2.1 Materials for AuNR Synthesis

Tetrachloroauric (III) acid trihydrate (HAuCl_4_·3H_2_O, ≥99.9% Sigma-Aldrich, St. Louis, MO, USA), cetyltrimethylammonium bromide (CTAB) (C_19_H_42_BrN, ≥97% Merck, Rahway, NJ, USA), sodium borohydride (NaBH_4_, 99.99% Aldrich, St. Louis, MO, USA), l-ascorbic acid (C_6_H_8_O_6_, AA, 99% Sigma, St. Louis, MO, USA), silver nitrate (AgNO_3_ 99.9%, Aldrich), and bidistilled H_2_O were used as received.

### 2.2 Synthesis of Gold Nanorods (AuNRs)

A two-step synthesis was performed, optimizing our previous procedure (see Figure 1).^19^ In the first step, the seed solution was prepared by adding 5 mL of a 0.2 M solution of CTAB to 5 mL of 0.0005 M HAuCl_4_. During the stirring, 600 μL of NaBH_4_ (0.01 M) was added and left in agitation for 5 min. The growth solution was prepared by adding 5 mL of CTAB (0.2 M), 5 mL of 0.001 M HAuCl_4_, and 200 μL of AgNO_3_ (0.004 M). Subsequently, 70 μL of AA at 0.078 M and 24 μL of seed solution were added, and all the solution was stirred for 20 min. For the purification, two centrifuges were made at 13,000 rpm for 15 min.

**Figure 1:**
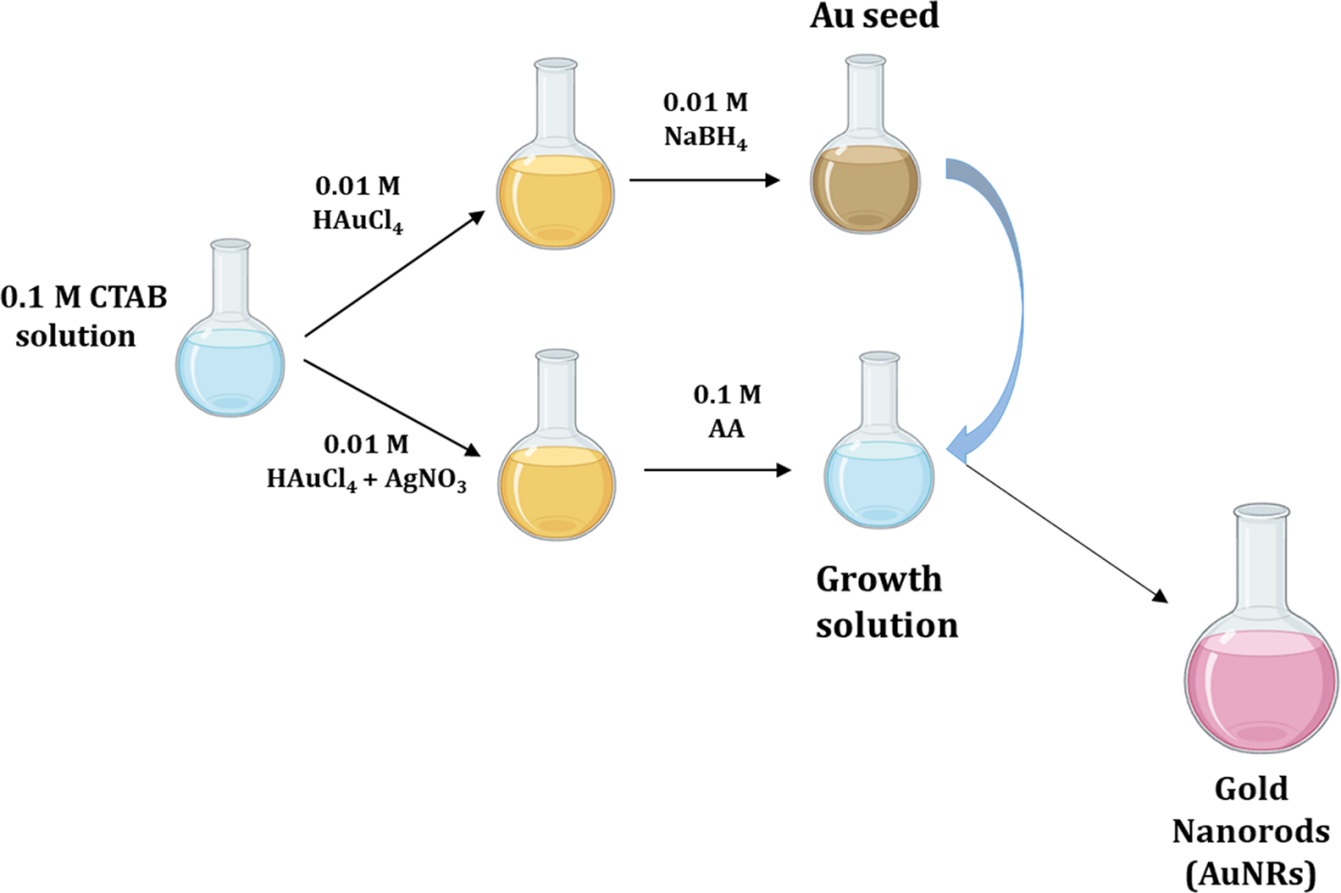
Scheme of the two-step synthesis of gold nanorods.^20^

### 2.3 TAT Preparation

#### 2.3.1 Materials for Peptide Synthesis

Rink Amide MBHA resin, 2-(1H-benzotriazol-1-yl)-1,1,3,3-tetramethyluronium hexafluorophosphate (HBTU), ethyl cyano­(hydroxyimino)­acetate (Oxyma Pure), anisole, acetic acid, dithiothreitol (DTT), Fmoc-7-aminoheptanoic acid, and all Fmoc-protected amino acids were purchased from Merck (Darmstadt, Germany). Triethylsilane (TES), *N*,*N*-dimethylformamide (DMF), dichloromethane (DCM), *N*,*N*-diisopropylethylamine (DIPEA), silver trifluoroacetate, and acetonitrile were purchased from Sigma-Aldrich (Merck KGaA, Darmstadt, Germany). Trifluoroacetic acid (TFA), piperidine, and ethyl ether were purchased from Biosolve (Leenderweg, Valkenswaard, The Netherlands). *N*-Methyl-2-pyrrolidone (NMP) was purchased from Iris Biotech GmbH (Marktredwitz, Germany).

#### 2.3.2 Cys­(Acm)-TAT Synthesis

The synthesis of Cys­(Acm)-TAT (sequence: CxRKKRRQRRR, where x is the 7-aminoheptanoic acid) was carried out via Fmoc chemistry using Rink Amide MBHA resin (0.62 mmol/g; scale 0.125 mmol) and the automated synthesizer Syro I (MultiSynTech, Witten, Germany). The side chain-protecting groups were Arg, Pbf; Cys, Acm; Gln, Trt; and Lys, Boc. The coupling reaction was performed using 5 equiv of amino acid, 5 equiv of HBTU/Oxima Pure in DMF, and 10 equiv of DIPEA in NMP. All the couplings were double. After the final Fmoc deprotection, the resin was washed with DCM and dried under vacuum for 1 h. The peptide was cleaved from solid support and at the same time deprotected from the side chain-protecting groups, apart from the Acm group, using a mixture of 2.5% H_2_O Milli-Q, 2.5% TES, and 95% TFA for 90 min under magnetic stirring. After cleavage, the resin was filtered, and the reaction mixture was concentrated using a rotary evaporator. Finally, crude Cys­(Acm)-TAT was precipitated with cold ethyl ether.

The crude Cys­(Acm)-TAT was purified by reverse phase-high performance liquid chromatography (RP-HPLC) at the following conditions: column Atlantis dC18 (10 μm, 100 Å, 10 × 250 mm, Waters); eluent A (0.05% TFA in H_2_O Milli-Q) and eluent B (0.05% TFA in CH_3_CN); gradient from 0% B to 7% B in 28 min and then from 7% B to 17% B in 20 min; flow rate, 4 mL/min; and detection at 214 nm. The chromatogram of purified Cys­(Acm)-TAT (Figure 2) was obtained using the following conditions: column Atlantis dC18; injection volume, 30 μL of 1 mg/mL peptide solution; flow rate, 1 mL/min; eluent A (0.05% TFA in H_2_O Milli-Q); eluent B (0.05% TFA in CH_3_CN); gradient from 5% B to 20% B in 30 min; and detection at 214 nm. The retention time resulted in 18.6 min, and the final peptide had a purity grade higher than 99%. The Cys­(Acm)-TAT identity was ascertained through mass spectrometry (Figure 3): experimental mass: 1639.83 Da and theoretical mass: 1640.069 Da (4800 MALDI-TOF/TOF TM instrument provided with 4000 Series Explorer TM software, Applied Biosystem/MDS Sciex, Framingham, MA, USA).

**Figure 2:**
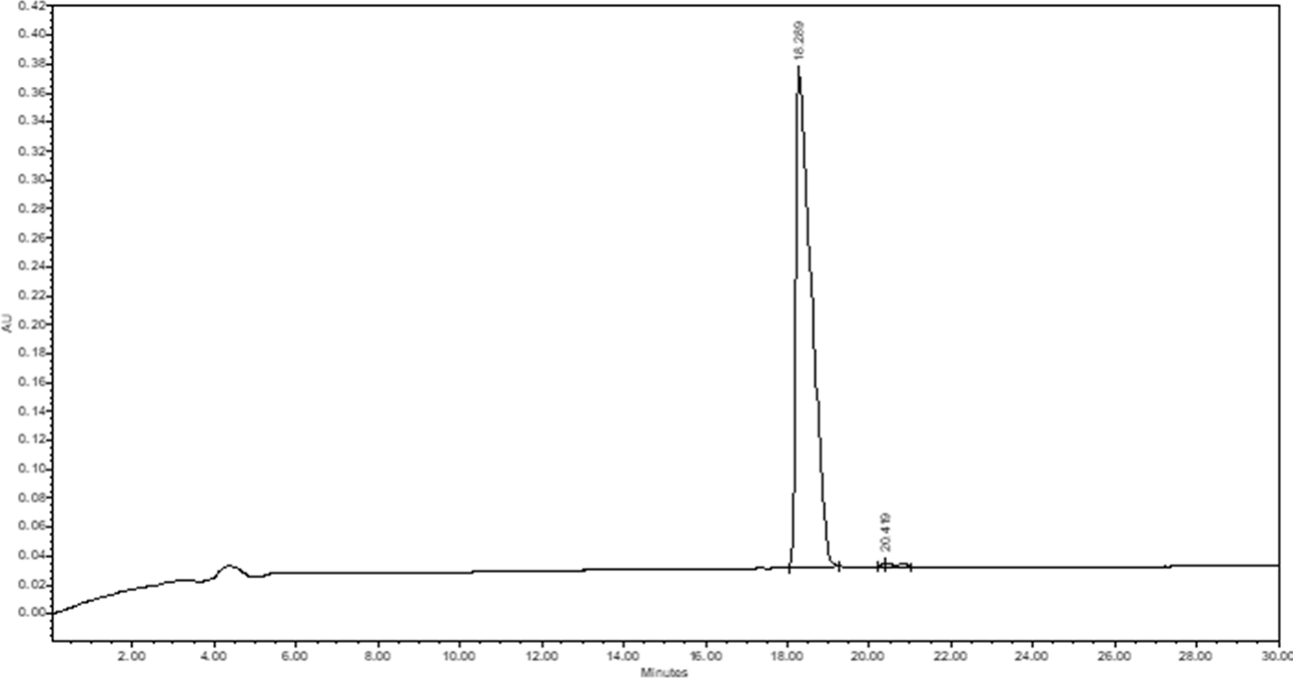
Analytical RP-HPLC chromatogram of purified Cys­(Acm)-TAT peptide. The analysis conditions were Atlantis dC18 (5 μm, 100 Å, 4.6 × 250 mm, Waters); injection volume, 30 μL of 1 mg/mL peptide solution; flow rate, 1 mL/min; eluent A (0.05% TFA in H2O Milli-Q) and eluent B (0.05% TFA in CH_3_CN); gradient, from 5%B to 20%B in 30 min; and detection at 214 nm. The retention time: 18.6 min.

**Figure 3:**
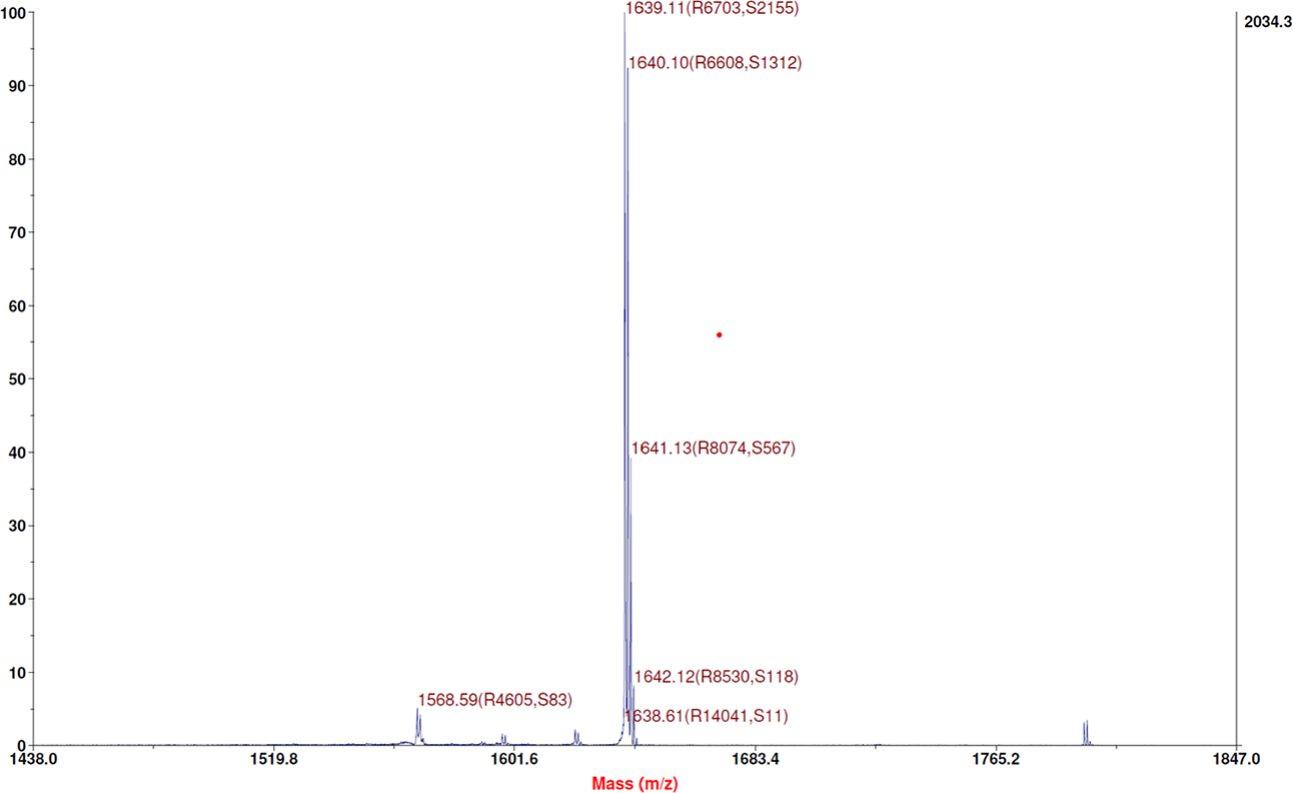
Mass Spectra of Cys­(Amc)-TAT. The identity of the pure peptide was ascertained by mass spectrometry; experimental mass: 1639.83 Da, theoretical mass: 1640.069 Da.

#### 2.3.3 Acm Group Deprotection

The Acm deprotection of cysteine was performed after the purification of Cys­(Acm)-TAT. The peptide was dissolved in TFA/anisole 99:1 (v/v) at a concentration of 1 mg/mL. A 100 equiv portion of silver trifluoroacetate was added, and the solution was maintained under magnetic stirring at 4 °C for 2 h. The mixture was then reduced to a small volume using a rotary evaporator, precipitated with cold ethyl ether, and dried under vacuum for 1 h. The peptide was then treated with 40 equiv of DTT in 1 M acetic acid for 3 h under magnetic stirring at room temperature. Then, the peptide was centrifuged, and the supernatant containing the deprotected peptide was filtered and analyzed in RP-HPLC (Figure 4) in the conditions described for purified Cys­(Acm)-TAT.

**Figure 4:**
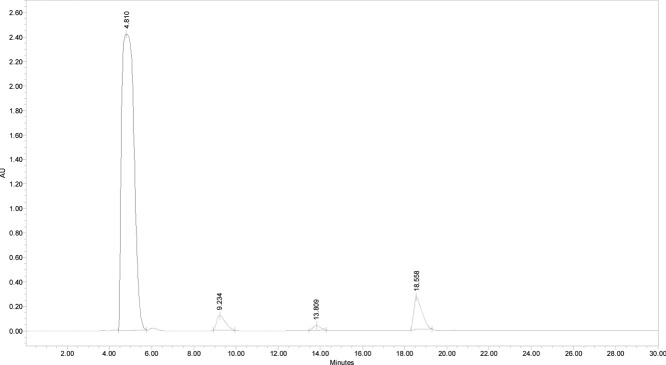
Analytical RP-HPLC chromatogram of crude Cys-TAT peptide. The analysis conditions were Atlantis dC18 (5 μm, 100 Å, 4.6 × 250 mm, Waters); injection volume, 50 μL of 1 mg/mL peptide solution; flow rate, 1 mL/min; eluent A (0.05% TFA in H_2_O Milli-Q) and eluent B (0.05% TFA in CH_3_CN); gradient, from 5%B to 20%B in 30 min; and detection at 214 nm.

Cys-TAT was purified following these conditions: column, Atlantis dC18; flow rate, 4 mL/min; eluent A (0.05% TFA in H_2_O Milli-Q) and eluent B (0.05% TFA in CH_3_CN); gradient, from 0% B to 15% B in 45 min; and detection at 214 nm. The analytical chromatogram of purified Cys-TAT (Figure 5) was obtained by using the same conditions reported above. The retention time resulted in 18.127 min, and the peptide purity grade was 98%. The identity of the purified peptide was ascertained by mass spectrometry (Figure 6), experimental mass: 1568.55 Da; theoretical mass: 1568.98 Da.

**Figure 5:**
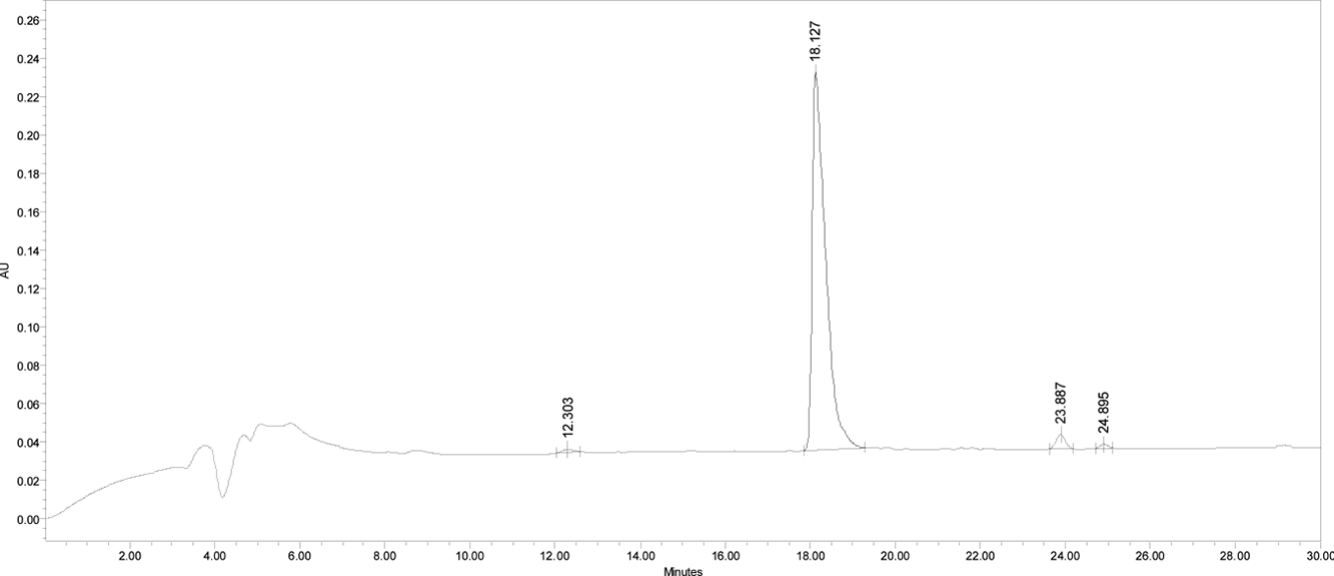
Analytical RP-HPLC chromatogram of pure Cys-TAT peptide. The analysis conditions were Atlantis dC18 (5 μm, 100 Å, 4.6 × 250 mm, Waters); injection volume, 50 μL of 1 mg/mL peptide solution; flow rate, 1 mL/min; eluent A (0.05% TFA in H_2_O Milli-Q) and eluent B (0.05% TFA in CH_3_CN); gradient, from 5%B to 20%B in 30 min; and detection at 214 nm. The retention time results 18.127 min.

**Figure 6:**
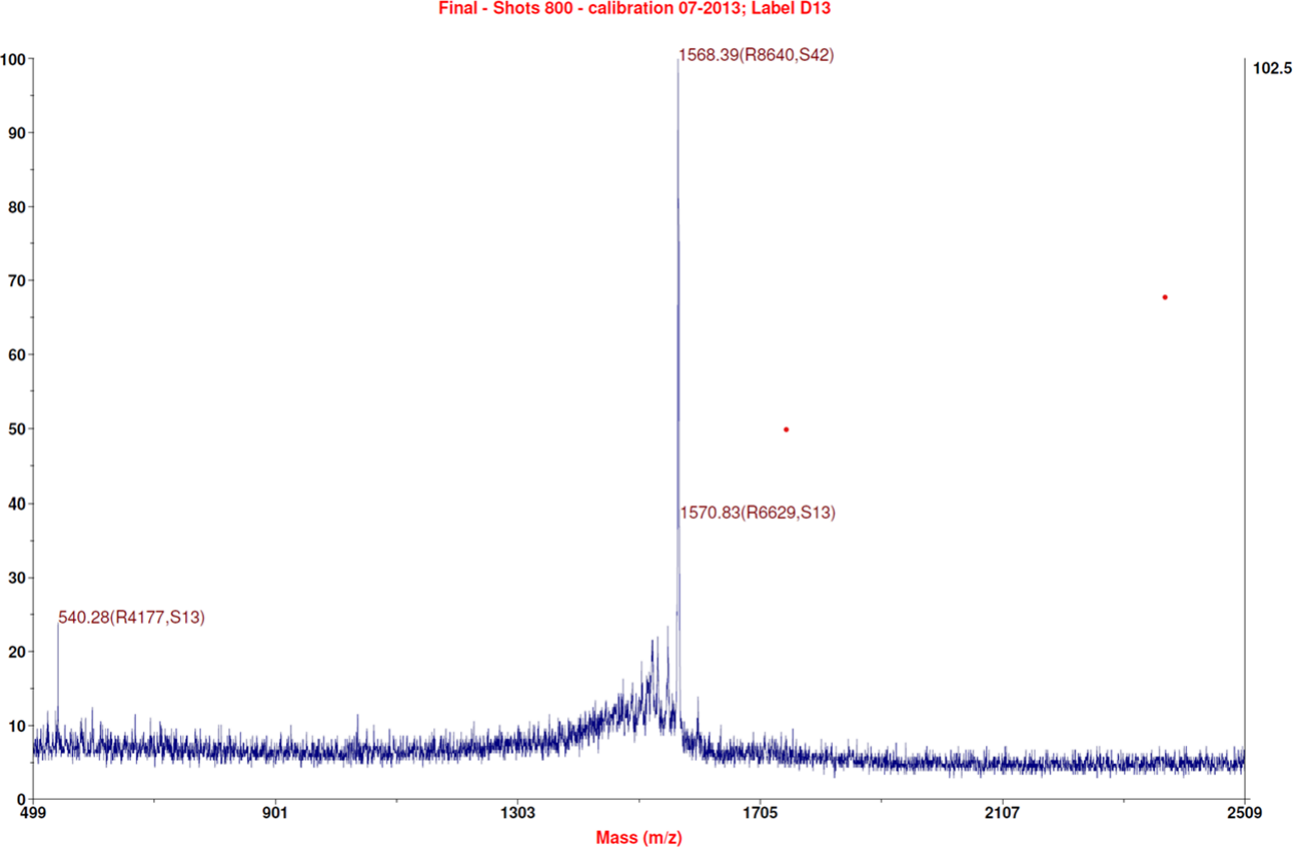
Mass spectra of Cys-TAT. The identity of the pure Cys-TAT peptide was ascertained by mass spectrometry; experimental mass: 1568.55 Da, theoretical mass: 1568.98 Da.

### 2.4 AuNRs-TAT Conjugation

For the TAT loading procedure, a protocol was performed in analogy to previous studies.^21,22^ Three loading experiments were performed. In the first case, a solution with a concentration of 6 × 10 ^–3^ g/tot in 5 mL of water of the non-protected peptide (TAT) was used, and 2 mL of that solution was added to 1 mg of AuNRs (2 mL of a solution concentrated at 0.5 mg/mL). The second experiment was performed by using the protective peptide, Acm-TAT, with the same concentration as the first one, 6 × 10^–3^ g/tot, and also 1 mg of AuNRs. In the last experiment, 2 mL of an 8 × 10^–3^ g/tot solution of non-protected peptide was added to 1 mg of AuNRs. All the experiments follow the same procedure, so all the solutions were stirred for 2 h at room temperature. To remove the non-loaded peptide, the solution was centrifuged for 15 min at 13,000 rpm.

### 2.5 Instrumentalization and Characterization

Both AuNRs and AuNRs-Cys-TAT were characterized by several different techniques.

UV–vis spectra were acquired in H_2_O by using a quartz cell with a Shimadzu 2401 PC UV–vis spectrophotometer in the wavelength range between 200 and 800 nm. For the measurement, a solution with a concentration of 0.5 mg/mL was used. The ζ potential and size distribution in water of AuNRs were investigated by means of Zetasizer Ultra Red, Malvern.^23,24^ Moreover, the stability over time of AuNR stock suspensions has been assessed by the measurement of zeta potential every week for one month.

Synchrotron radiation-induced X-ray photoelectron spectroscopy (SR-XPS) experiments were carried out at the SuperESCA beamline at the ELETTRA synchrotron facility of Trieste (Italy). XPS data were collected in fixed analyzer transmission mode (pass energy = 10.00 eV), with the monochromator entrance and exit slits optimized at 30 and 20 μm, respectively. For the C 1s, N 1s, O 1s, and Ag 3d spectral regions, a photon energy of 600 eV was used; S 2p, Br 3d, and Au 4f core levels were measured with PE = 260 eV to maximize signal intensity and resolution. The energy resolution was about Δ*E* = 0.25 eV for all signals. Calibration of the energy scale was made referencing the spectra collected with 600 eV PE to the C 1s core-level signal of aliphatic carbons, found at 285.00 eV, and the signals for which the PE was 260 eV to the Au 4f_7/2_ component of metallic gold at 83.7 eV, for both samples. SR-XPS measurements were carried out in the solid state by drop-casting a drop of aqueous suspension onto TiO_2_/Si­(111) wafer surfaces. Curve-fitting analysis of the C 1s, N 1s, O 1s, Ag 3d, Au 4f, S 2p, and Br 3d spectra was done using Gaussian curves as fitting functions, after subtraction of a polynomial background. The S 2p_3/2,1/2_ doublets were fitted using the same full width at half-maximum (fwhm) for both components and a spin–orbit splitting of 1.2 and a branching ratio (2p_3/2_/2p_1/2_) of 2/1. The Au 4f_7/2,5/2_ doublets were fitted using fwhm for both components, a spin–orbit splitting of 3.7 and a branching ratio of 4/3. The Br 3d_5/2,3/2_ doublets were fitted using fwhm for both components with a spin–orbit splitting of 1.04 ^25^ and a branching ratio of 3/2. The Ag 3d_5/2,3/2_ doublets were fitted using fwhm for both components, a spin–orbit splitting of 6.0 and a branching ratio of 3/2. When several different species were identified in a spectrum, the same fwhm value was set for all individual photoemission bands.

Near-edge X-ray absorption fine structure (NEXAFS) spectra were acquired at the BEAR beamline (bending magnet for emission absorption and reflectivity) and installed at the left exit of the 8.1 exit of the ELETTRA synchrotron facility. BEAR uses a bending magnet as a source and beamline optics delivering photons from 5 eV up to about 1600 eV. In our experiments, ammeters were used to measure the drain current from the sample. Samples of both the pristine cys-TAT peptide and of Au-NRs-cys-TAT nanorods were prepared as thin films by casting from aqueous solution onto Au/Si(111) wafer surfaces. C K-, N K-, and O K-edge spectra were recorded at a grazing (20°) incidence angle of the impinging photon beam with respect to the sample surface in order to maximize signal intensity. In addition, our carbon and nitrogen K-edge spectra have been further calibrated using the resonance at 288.70 eV, assigned to the C=O 1s→π* transition, and the resonance at 402.00 eV, assigned to the 1s→π* transition of the peptide bonds, respectively. The raw spectra were normalized to the incident photon flux by dividing the sample spectrum by the spectrum collected on a freshly sputtered gold surface. The spectra were finally normalized by subtracting a straight line that fits the part of the spectrum below the edge and setting to 1 the value at 330.00, 430.00, and 560 eV for the C K-, N K-, and O K-edge spectra, respectively.

Reflection–absorption infrared spectroscopy (RAIRS) measurements were carried out on thin films of cys-TAT and of Au-NRs-cys-TAT by casting from aqueous solution onto Au/Si(111) wafers. IR spectra were recorded in the 400–4000 cm^–1^ wavenumber range (resolution 1 cm^–1^) by means of a VECTOR 22 (Bruker) FT-IR interferometer equipped with a Specac P/N 19 650 series monolayer/grazing angle accessory and with a DTGS detector; the incidence angle of the IR beam with respect to the sample surface was 20°.

X-ray absorption spectroscopy (XAS) measurements were carried out at the LISA-BM08 beamline^26^ at ESRF (European Synchrotron Radiation Facility), probing the Au LIII- and Ag K-edges of the TAT-functionalized gold nanorods. The AuNRs-TAT sample was prepared by starting by concentrating a solution of gold nanorods via centrifugation, remaining with just a few droplets of suspended nanorods. A suitable amount of cellulose was added, and the sample was then dried in low-vacuum conditions, mixed, and pressed to obtain a homogeneous pellet suitable for handling (15 mm ⌀). The beamline optics featured a Si(311) double crystal monochromator equipped with harmonic rejection mirrors (Pt coating). XAS spectra were collected in fluorescence geometry using a High Purity Germanium (HP-Ge) multidetector (13 elements, ORTEC) under low pressure conditions and at a temperature of 80 K due to cooling with liquid nitrogen. For each edge probed, a metallic foil of the same material as the absorber was placed in vacuum after the sample and acquired in transmission geometry using two gas-filled ionization chambers I1 and I2, respectively, before and after the reference material. The absorption signals of the sample and reference were calculated, respectively, as $\alpha_{\exp}=\sum_{i}\frac{I_{fi}}{I_{0}}$ and $\alpha_{ref}=\ln\frac{I_{1}}{I_{2}}$ , where ∑*I*_f_ is the sum over all the detector channel elements (except those with a lower signal-to-noise ratio) and I_0_ is the intensity measured in a first ionization chamber (prior to the sample). The absorption spectrum of a pure reference foil placed after the sample was used to check the energy calibration during data collection and eventually align the energy scale of the spectra. Multiple spectra, respectively, 4 at the Au L_III_ and 9 at the Ag K-edge, were measured, checked for energy calibration, and averaged to obtain data statistics suitable for quantitative analysis. To extract EXAFS structural signal χ_exp_, the experimental spectra α_exp_ were treated according to standard procedures^27^ including linear pre-edge subtraction (α^′^ = α_exp_ – α_pre_), bare atomic background (α_b_) subtraction, and normalization, to extract EXAFS structural signals: $\chi_{\exp}\left(k\right)=\frac{\left(\alpha^{\prime }-\alpha_{b}\right)}{\alpha_{b}}$ . The edge energy *E*_0_, the origin of the photoelectron wavenumber $k={\mathrm{h̵}}^{-1\sqrt{2m_{e}\left(E-E_{0}\right)}}$ (*m*_e_ being the electron mass), was defined as the first inflection point (maximum of the first derivative) of the spectra. The quantitative analysis of the EXAFS signals was carried out by fitting the k^w^-weighted theoretical curves k^w^χ_th_ to the raw experimental data *k* ^w^χ_exp_, applying a nonlinear least-squares procedure implemented in the program FiteEXA.^27^ The theoretical curves χ_th_(k) were calculated as a sum of partial contributions χ_*i*_, calculated using a Gaussian pair distribution function model and the standard EXAFS formula,^28,29^ with a Gaussian disorder model. The theoretical photoelectron scattering amplitude and phase functions were calculated using the FEFF8 program.^30^

Transmission electron microscopy (TEM) images were acquired with a probe-corrected JEOL JEM ARM200F microscope operated at 200 kV equipped with a Gatan Ultrascan CCD camera. A 10 μL portion of samples was drop-casted on Formvar/carbon-supported copper grids. Grids were left to dry at room temperature, and then they were examined.

## 3 Results and Discussion

### 3.1 Synthesis and Conjugation of AuNRs-TAT

The synthesis of the TAT peptide did not present particular problems. The purified Cys­(Acm)-TAT was 29.58% of the crude Cys­(Acm)-TAT. According to previous article,^19^ AuNRs with an aspect ratio of 3.2 were obtained. The polydispersity was low, and the particles resulted in being homogeneous. The AuNRs were checked by UV–visible spectroscopy by obtaining the typical two plasmonic peaks, the transverse at 520 and the longitudinal at 770 nm (Figure 7a, green line). The second step was to confirm the presence of Cys-TAT on AuNRs, so they were analyzed and compared with the AuNRs only and the Cys-TAT, and the presence of the Cys-TAT was confirmed by the presence of the typical peak at 260 nm (Figure 7a,b; see the pink bar).

**Figure 7:**
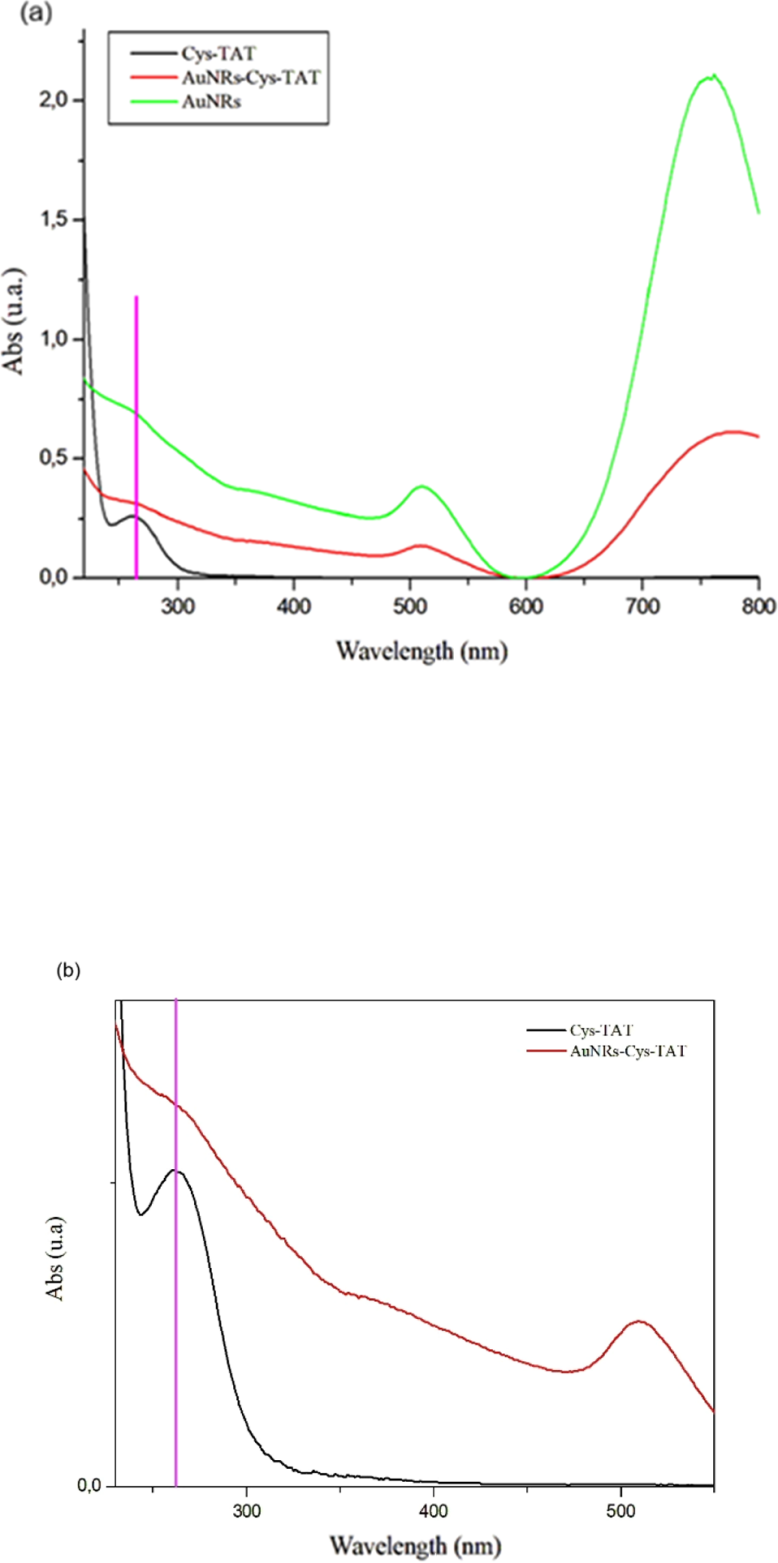
(a) Comparison between the UV–visible spectra of Cys-TAT peptide alone, AuNRs alone, and combined AuNRs-Cys-TAT. The pink line at 260 nm shows the cys-TAT peptide contribution; (b) the contribution of the cys-TAT peptide can be better appreciated in this enlargement.

DLS measurement performed on AuNRs in Milli-Q water showed a hydrodynamic diameter of 8.43 ± 0.1 nm and a polydispersity index (PDI) of 0.647. The difference between DLS and FESEM data on AuNR size and the high value of PDI is due to the different physical properties measured by these two techniques (hydrodynamic diameter versus projected diameter) and to the morphology of the AuNRs, which is far from the spherical shape on which DLS is based to determine these characteristics. Electron microscopy allows one to determine the primary size of particles, whereas DLS measures the time-dependent fluctuations in scattering intensity due to constructive and destructive interference resulting from the relative Brownian movements of the NPs. Through the autocorrelation function and subsequent calculation of the exponential decay, it determines the hydrodynamic diameter that represents the diameter of an equivalent rigid sphere that diffuses at the same rate as the analyte.^31^ The time-dependent position or velocity of the suspended particles affects frequency shifts resulting from light scattered by the suspended particles, leading to misleading results for hydrodynamic diameter, polydispersion, and aggregation state for nonspherical NPs.

AuNRs show positive surface charge and high stability over one month for AuNRs in Milli-Q water as indicated by the values of zeta potential greater than 30 mV (Figure 8).

**Figure 8:**
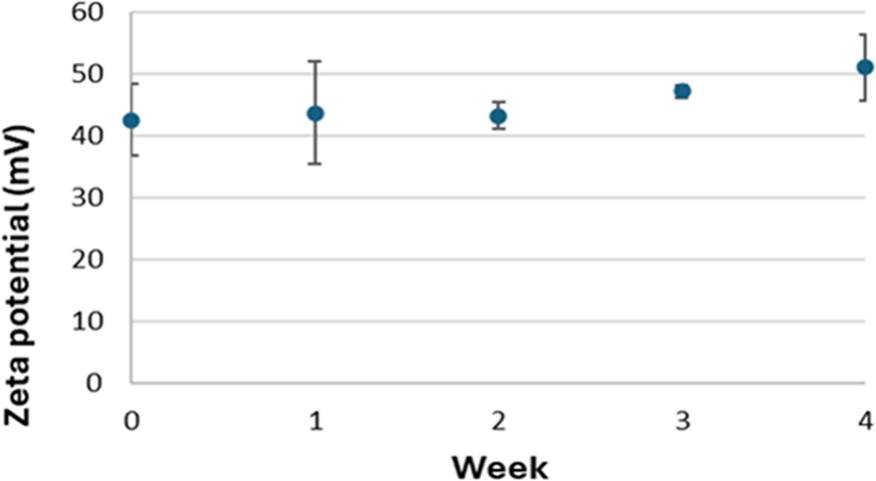
Time trend of AuNR’s zeta potential.

The AuNRs-Cys-TAT show a great state of aggregation due to the presence of the cysteine’s thiol group, unlike the AuNRs-Acm-TAT that show a minor aggregation due to the presence of the protection group, Acm, present on the cysteine. Moreover, the presence of the Acm group prevents the possible aggregation of the peptide by the creation of sulfide bridges between the cysteine, increasing the possibility of loading on the AuNR surface. This is confirmed by the TEM analysis.

Conventional TEM (CTEM) images for AuNRs with Acm-TAT and Cys-TAT are reported in Figures 9A and 9B, showing nanorods and some nanoparticles, mostly spherical. For both samples, nanorods have a length ranging from 18 to 52 nm and a thickness from 9 to 12 nm. The size of the particles varies from 9 to 28 nm. High-resolution TEM (HRTEM) images (Figures 9C and 9D) demonstrate that the nanoparticles are well crystallized. In these images, lattice fringes of 2.3 Å and 2.0 Å corresponding to (111) and (200) planes of Au crystallized in a cubic structure are well observed.

**Figure 9:**
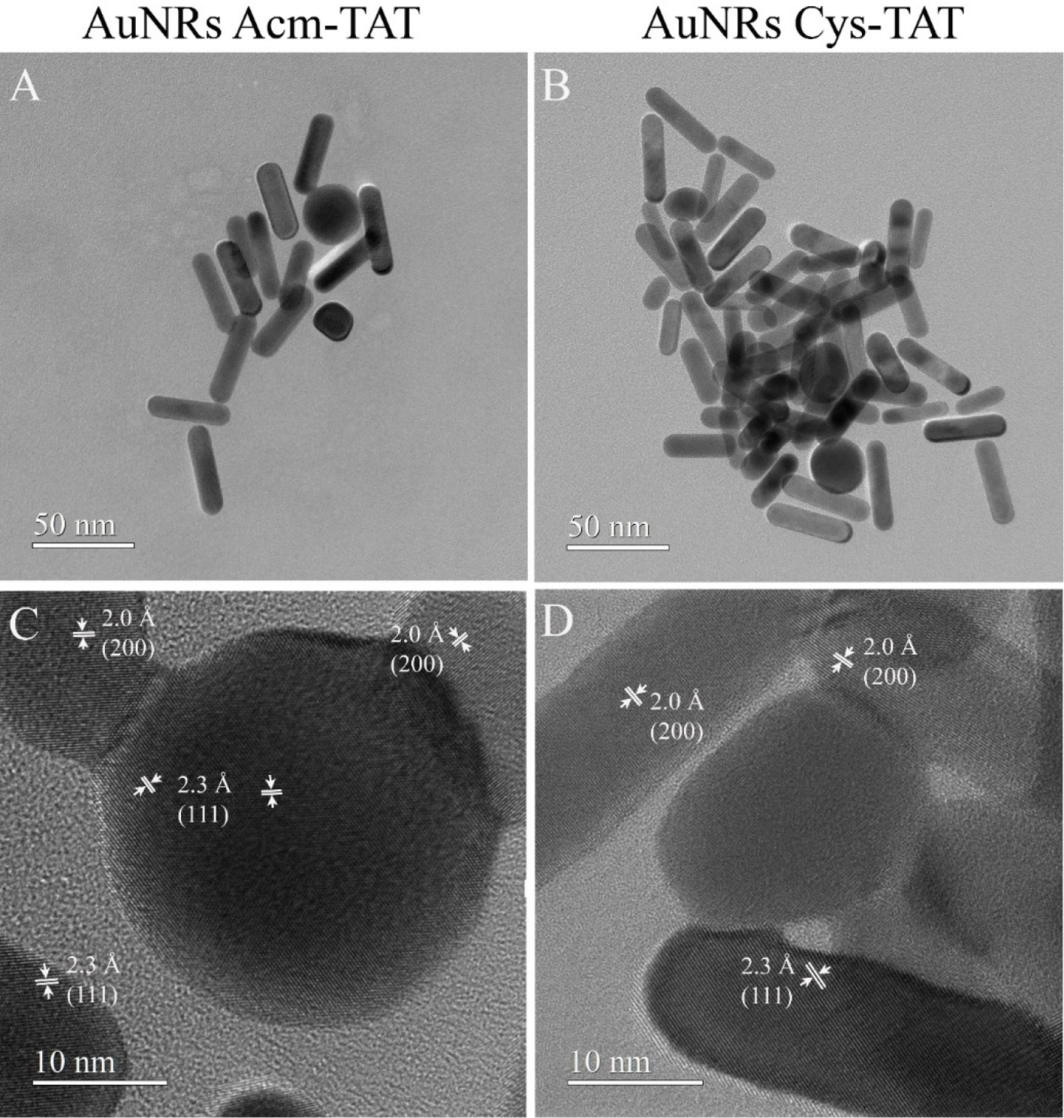
CTEM (A,B) and HRTEM (C,D) images of AuNRs with Acm-TAT and Cys-TAT.

### 3.2 Structural Characterizations

#### 3.2.1 High-Resolution X-ray Photoelectron Spectroscopy Results: Molecular and Electronic Structure at the Peptides/Gold Nanorod Interface

The XPS data analysis, carried out in solid-state samples of pure-pristine TAT-x-cys and AuNRs-Cys-TAT-x-cys conjugate deposited as thin films from aqueous solution onto TiO_2_/Si­(111) substrates, allowed the successful anchoring of the peptide in both forms onto the AuNR surface and their molecular structure stability upon interaction with the nanoparticles. All measured spectra and spectral components individuated by applying a peak fitting procedure are shown in Figure 10 and discussed in detail in the following. All binding energy (BE), full width at half-maximum (fwhm), atomic percentage values, and proposed assignments are reported in Table S1.

**Figure 10:**
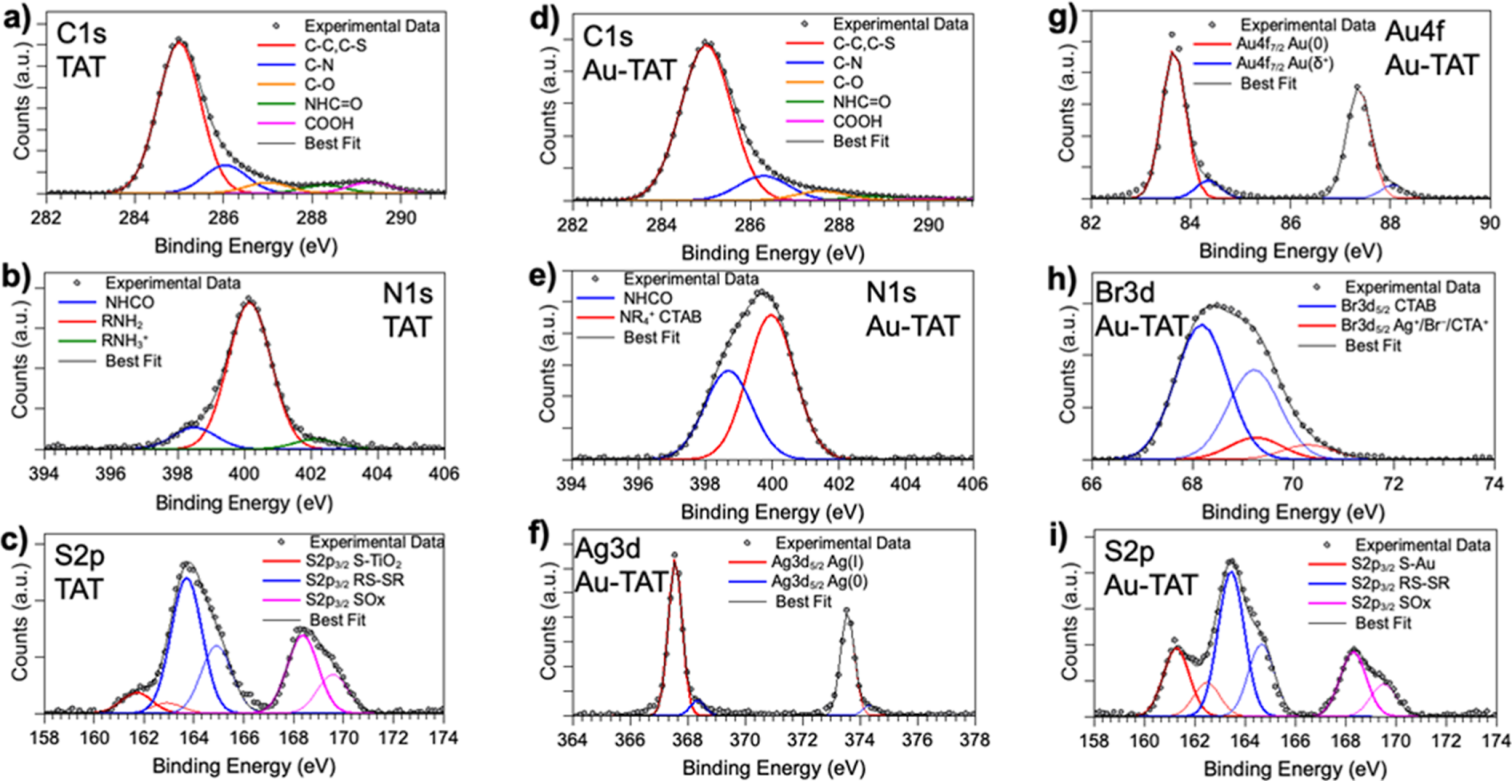
SR-XPS spectra collected on sample TAT-x-cys at (a) C 1s, (b) N 1s, and (c) S 2p core levels and on sample AuNRs-Cys-TAT-x-cys (namely, Au-TAT in the figure) at (d) C 1s, (e) N 1s, (f) Ag 3d, (g) Au 4f, (h) Br 3d, and (i) S 2p core levels.

C 1s spectra collected on TAT-x-cys and AuNRs-Cys-TAT-x-cys are reported in Figure 10a,d. The same spectral components are observed for the peptide sample and the functionalized nanoparticles AuNRs-Cys-TAT-x-cys: C–C and C–S groups at 285.00 eV BE, C–N at 286.3 eV BE, C–O at about 287 eV BE, NHCO (amide-like C atoms) at nearly 288 eV BE, and COOH groups at 289–290 eV BE.^32^ It is noteworthy that the atomic percentage of the C–C component is higher in the functionalized nanorods, as expected due to the presence of CTAB. N 1s spectra (Figure 10b,e) show two main components arising, respectively, from amide (398.5 eV) and amine-like N atoms (400.2 eV BE) and a small feature due to positively charged amine groups (402 eV) for TAT-x-cys, as usually observed for amino acids;^33^ for AuNRs-Cys-TAT-x-cys, the peak fitting revealed larger components due to the increased surface roughness of the sample, and it was not possible to distinguish the amine and amide-like signals that appear superimposed at about 398.7 eV BE. The intense peak at about 400 eV BE is indicative of NR_4_^+^ tertiary amines of CTAB.^25^ S 2p spectra (Figure 10c,i) are composed of several components and are very similar for pristine TAT-x-cys and AuNRs-Cys-TAT-x-cys, suggesting a very good chemical stability of the TAT-x-cys peptide upon anchoring to the AuNR surface. The analysis of S 2p spectral components BE and intensities will shed some light on the chemical interaction of peptides/AuNRs. First of all, the spin–orbit pair at lower BE is indicative of S atoms of thiol moieties chemically bonded to metals;^34^ in pristine TAT-x-cys, this is a very low intensity signal (about 8% atomic percentage) attributed to the interaction of the peptide with the TiO_2_ substrate in the thin film (BE S 2p3/2 = 161.8 eV), whereas in AuNRs-Cys-TAT, it is shifted at lower BE values (BE S 2p_3/2_ = 161.2 eV), as expected for thiol end groups covalently bonded to the gold atoms at the AuNR surface,^34^ and of higher intensity (24.5% atomic percentage when the S 2p signal is measured at 260 eV PE). It is noteworthy that in the S 2p spectrum measured with a PE = 600 eV, i.e., increasing the sampling depth, the atomic percentage of the RS-Au component increases by about 32%. This is a further indication for the RS-Au covalent bonding, arising at the AuNRs/peptide interface. As for the higher BE components, in both TAT-x-cys and AuNRs-Cys-TAT-x-cys, there is a main feature assigned to unreacted thiol moieties and/or disulfides (RSH, RS-SR, BE S 2p_3/2_ = 163.5 eV), as expected due to the high reactivity of thiol moieties, and a percentage of oxidized sulfur species, also expected due to the TAT exposure to air and water for sample deposition and anchoring procedure (BE S 2p_3/2_ = 168.3 eV, 23% in AuNRs-Cys-TAT-x-cys and 33% in pristine TAT-x-cys). Au 4f, Br 3d, and Ag 3d core-level spectra collected on AuNRs-Cys-TAT-x-cys are, respectively, reported in Figure 10g,h,f, and they show all the components expected for AuNRs, as already extensively discussed by some of us in a previous publication.^25^ In conclusion, SR-XPS data analysis allowed us to assess the molecular stability of the TAT-x-cys peptide upon interaction with the AuNRs, as well as to probe the formation of a covalent chemical bond between the thiol moieties of TAT-x-cys and the gold atom at the AuNR surface. At the same time, the stability of AuNRs was also assessed by the reproducibility of Au 4f, Br 3d, and Ag 3d spectra with respect to previously published data collected on AuNRs stabilized by CTAB and secondary surfactants.

#### 3.2.2 NEXAFS

The NEXAFS spectra recorded at the C K-, N K-, and O K-edges for the pristine cys-TAT peptide and for the AuNRs-cys -TAT nanorods are shown in Figure 11. A summary of the main peak positions and assignments is also shown in Table S2.

**Figure 11:**
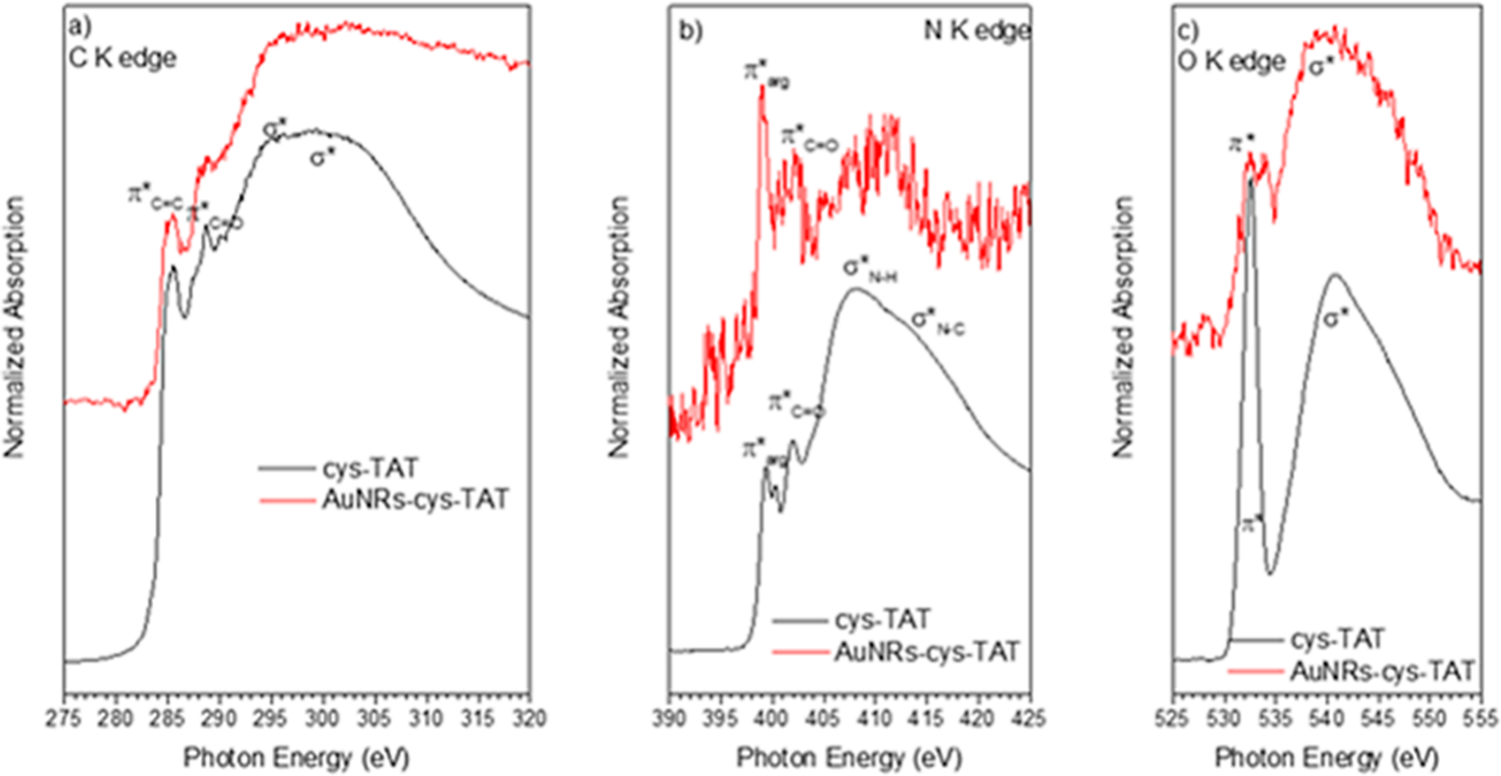
C K-edge (a), N K-edge (b), and O K-edge (c) NEXAFS spectra of pristine cys-TAT (black line) and AuNRs-cys-TAT (red line).

In the C K-edge spectrum of peptide cys-TAT (Figure 11a, black line), two C 1s→π * resonances are detected below the edge, located at 285.4 and 288.7 eV (see Table S2), in increasing photon energy order. The second π * resonance, located at 288.7 eV, is typical of C=O bonds related to the amide functions of the peptide backbone of cys-TAT, as already detected for similar peptide systems.^35,36^ The peak at 285.4 eV is related to transitions into antibonding C=C orbitals. Above the edge, broad σ* resonances related to C–C and C=O molecular orbitals are evidenced (Table S2).

The N K-edge spectrum of cys-TAT (Figure 11b, black line) also shows two N 1s→π* resonances; the peak located at 402 eV can be assigned to 1s→π* transitions of the peptide bonds, and the resonance at 399.5 eV can be assigned to 1s→π* transitions arising from the C=N bonds of the guanidine function of arginine, which is the principal component of the cys-TAT peptide.^18^ Above the edge, two N 1s→σ* resonances, arising from N–H and C–N bonds, are detected. Finally, the O K-edge spectrum of cys-TAT, displayed in Figure 11c, red line, shows a single O 1s→π* resonance due to the C=O bonds of the peptide backbone, with a related broad O 1s→σ* resonance above the edge. The NEXAFS spectra recorded at the C, N, and O K-edges for AuNRs-cys-TAT (Figure 11a–c red lines) show the same peaks, exactly in the same position, as the corresponding spectra of the pristine cys-TAT peptide. The low intensity of the signal arising from the peptide overlayer chemisorbed on the AuNR surface reduces the signal-to-noise ratio, generating noisy spectra, particularly for the N K-edge. Nevertheless, it is evident that the positions of the π* resonances in the measured spectra are the same for cys-TAT and AuNRs-cys-TAT, suggesting that the molecular structure of the TAT peptide is not altered by chemisorption on the AuNR surface.

#### 3.2.3 FTIR

The 4000–2400 cm^–1^ region of the FTIR spectrum of cys-TAT (Figure 12, black line) is dominated by the superimposition of the O–H and N–H stretching vibrations, producing an intense and broad band located at about 3500 cm^–1^; low-intensity peaks located at 2980–2910 cm^–1^ are related to aliphatic C–H stretching vibrations. The stretching vibration of the S–H bond of the cysteine moiety (labeled ν_S–H_ in the FTIR figure) is located at 2515 cm^–1^.

**Figure 12:**
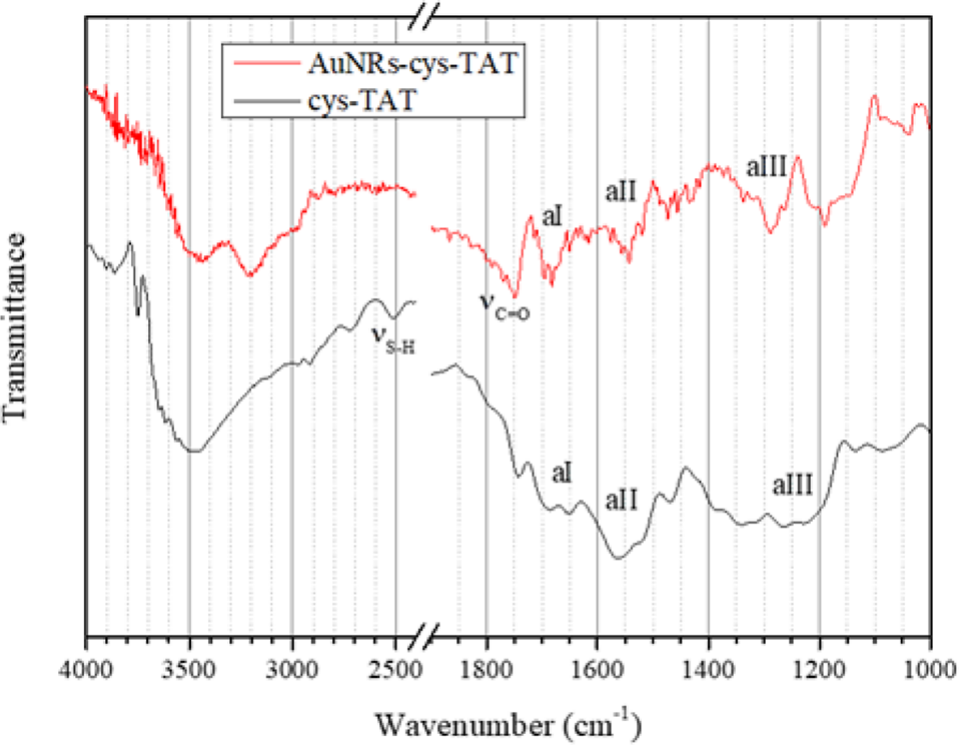
FTIR spectra of cys-TAT (black line) and AuNRs-cys-TAT (red line) in the 4000–2400 and 1900–1000 cm^–1^ regions.

The 1700–1400 cm^–1^ region of the spectrum shows the superimposition of a large number of peaks. The IR spectra of peptides always show three peaks typical of the amide functions of the peptide backbone, i.e., the amide I band (labeled aI in the FTIR figure), related to C=O stretching and located typically between 1700 and 1600 cm^–1^; the amide II band (aII), due to N–H bending and located between 1600 and 1500 cm^–1^; and finally the amide III band (aIII), related to C–N stretching and N–H deformations, located between 1350 and 1250 cm^–1^. The shape and position of the amide I band are related to the secondary structure of the peptide chain.^37^ Moreover, the cys-TAT peptide is rich in arginine, and the guanidine function in the pending group of this amino acid produces an intense C=N stretching peak located at 1666–1662 cm^–1^ and a N–H bending band around 1554–1537 cm^–1^; the position of this second peak can be affected by nitrogen protonation and hydrogen bonds.^38,39^ Superimposition of all of these peaks creates the complex pattern in the cys-TAT spectrum.

In the FTIR spectrum of AuNRs-cys-TAT (red line), the peak at 2515 cm^–1^ related to S–H stretching of the thiol group of cysteine disappears, indicating that the covalent attachment of cys-TAT to the AuNR surface actually takes place through the thiol moiety. The amide I, II, and III bands, on the other hand, are clearly visible around 1670, 1540, and 1280 cm^–1^, respectively, proving the effectiveness of the functionalization of the AuNR surface. In the high wavenumber region, a new peak appears around 3200 cm^–1^, while the intensity of the peak at 1750 cm^–1^(ν_C=O_), slightly visible in the spectrum of cys-TAT, increases. Both effects could be related to residues of ascorbic acid (AA) on the AuNR surface.^38^

#### 3.2.4 X-ray Absorption Spectroscopy Analysis

XAS measurements on gold nanorods functionalized with the peptide TAT (AuNRs-TAT) were performed at the Au LIII and Ag K edges at the LISA (BM08) beamline of the ESRF.^26^ XAS spectra were analyzed in both the XANES and EXAFS regions, providing complementary information on the local ordering around the average absorber. The XANES technique features the analysis of the absorption spectra from a few eV before the edge up to 200 nm after *E*_0_, providing information on the average valence state of the absorber, the density of empty states near the Fermi level, and the average coordination symmetry around the absorber.^40^ Analysis of the EXAFS data provides further details on the average local atomic coordination around the absorber with average distances, multiplicities, and mean squared relative displacement (MSRD) of the neighboring shells.^28^ XANES analysis of the Au LIII-edge is shown in Figure 13, and the sample showed an absorption edge *E*_0_ at 11,919 eV, consistent with the presence of metallic Au(0) gold.

**Figure 13:**
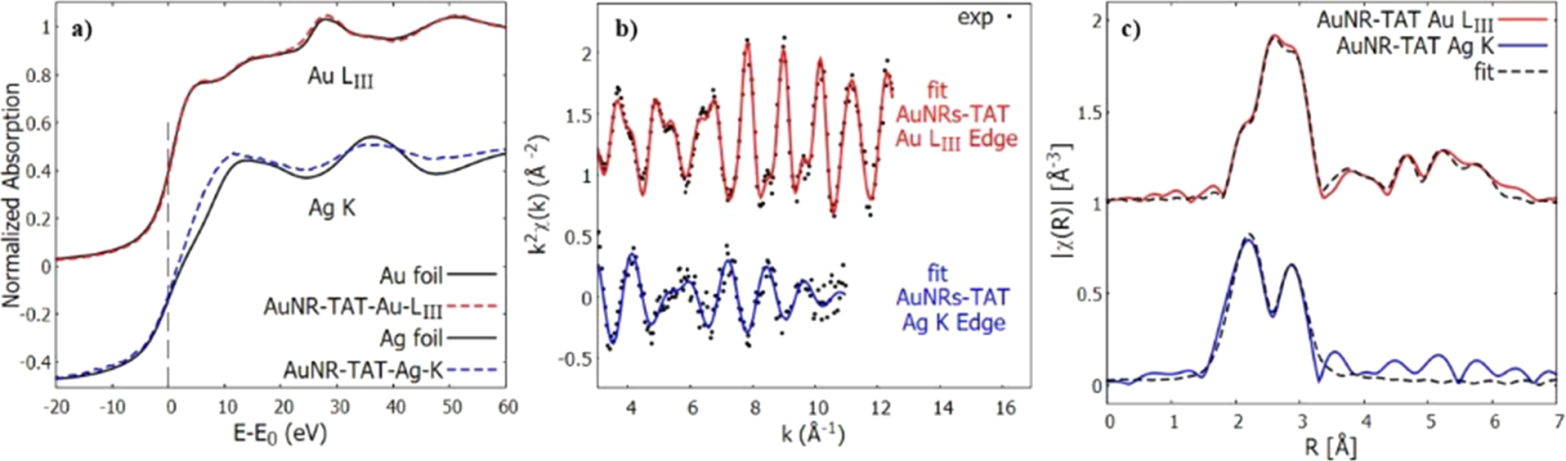
(a) Normalized XANES spectra of AuNRs-TAT at the Au LIII (red line) and Ag K (blue line) edges. Reference metallic foils (black dotted lines) are shown for comparison. (b) Experimental EXAFS data (black dots) and best-fit results in K­[Å-1] space for AuNRs-TAT at the AuLIII (red line) and Ag K (blue line) edges. (c) Experimental data (black dotted line) and best-fit results for Au LIII (red line) and Ag K (blue line) edges in R­[Å] space (|FT| of *kx* χ­(*k*), with *x* = 2 for Au and *x* = 1 for Ag).

The first shoulder right after the edge, at about 11,920 eV, is referred to as the “white line” region for gold and is related to photoelectron transitions from the 2p_3/2_ state to the 5d state; the intensity of the white line is proportional to the density of free states in the 5d valence.^41^ The system also shows extreme similarity to the spectrum of the reference gold material, which is a foil of bulk fcc-structured gold. As observed before^25^ on the pristine gold nanorod, the nanorods’ structure was not impacted by the functionalization procedure, showing a bulk-like environment of gold coherent with gold nanorods of relatively large dimensions, with negligible contributions from the surface. The Ag K-edge of AuNRs-TAT instead shows clear differences from that of the reference material (a metallic foil of fcc silver), revealing the presence of a mixture of metallic Ag(0) and oxidized Ag­(I) silver species. A metallic absorption edge *E*_0_ at 25,514 eV is followed by an increased intensity right after the edge, in the “white line” region, which is characteristic of an oxidized state of silver Ag­(I). The post-edge region then resumes a resemblance of metallic behavior with similar oscillations except for a dampening factor.

Quantitative EXAFS analysis of the Au LIII-edge confirms the presence of a bulk-like environment for gold nanorods, with experimental data refined using the crystallographic structure of gold (from the Crystallography Open Database^42,43^ ). The cubic face-centered cubic (fcc) structure helped to identify and fit scattering components up to the fourth shell of coordination. More in detail, 4 single scattering (SS) contributions (Au–Au1 with coordination number (C.N.) 12 at a distance of 2.86 Å, Au–Au2 with C.N. 6 at a distance of 4.05 Å, Au–Au3 with C.N. 24 at a distance of 4.99 Å, and Au–Au4 with C.N. 12 at a distance of 5.73 Å) and 3 multiple scattering (MS) contributions (Au–Au1–Au1 with C.N. 48 at a distance of 4.30 Å and Au–Au1–Au4 and Au–Au1–Au4–Au1 with relative C.N. of 24 and 12 at a distance of 5.73 Å, respectively) were needed to fit the experimental signal. A comparison between experimental data *k*_2_χ­(*k*) and the best-fit result is shown in Figure 13b, where the theoretical χth­(*k*) were calculated as a sum of partial contributions χ_*i*_, calculated using a Gaussian pair distribution function model and the standard EXAFS formula^28^ with a Gaussian disorder model as detailed in the “Materials and Methods” section. Table 1a presents a detailed comparison between experimental data and the best-fit result, showing also the contributions of each shell to the fit as well as residuals. After a trial-and-error procedure to reduce the number of variables in the fit, a set of constraints was employed: the coordination numbers of each scattering path included were fixed to their theoretical value for the bulk structure of gold, refining interatomic distances R­[Å] and mean-squared relative displacement σ2­[Å2]. Additionally, the distances of Au–Au4, Au–Au1–Au4, and Au–Au1–Au4–Au1 were refined as one since they share an identical value. Lastly, the mean square displacement σ2 of the triple scattering Au–Au1–Au4 was fixed as the averaged value of the displacements of the single scattering Au–Au4 and the quadruple scattering Au–Au1–Au4–Au1. The EXAFS analysis best-fit results, as reported in Table 1b, showed a consistent shortening in the distances obtained compared to the theoretical values, coherent with a slight compression of the gold fcc lattice of ∼0.6% that could be due to thermal contraction since measurements were taken at 80 K.

**Table 1:**
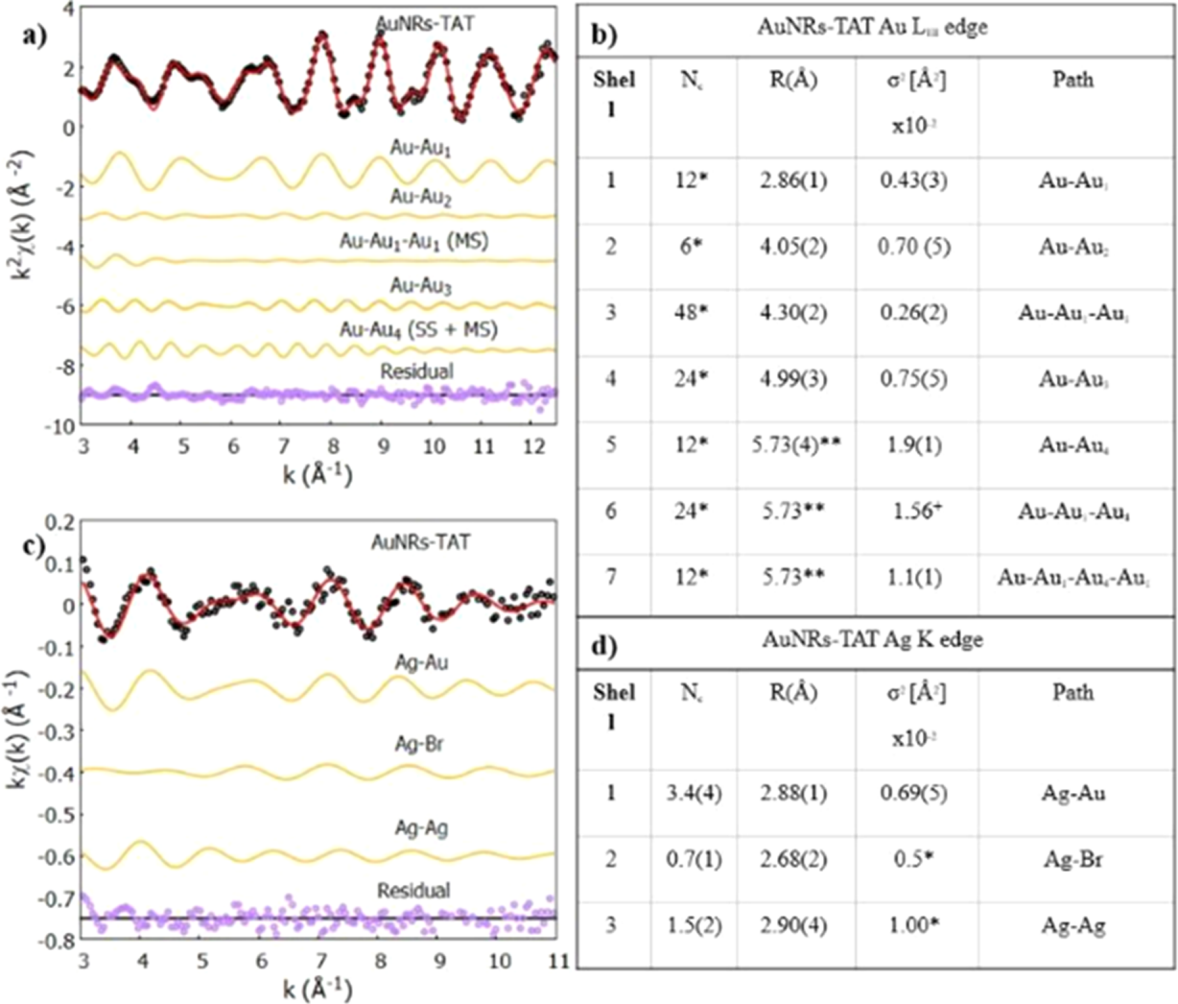
Comparison between Experimental (Black) and Best-Fit (Red) Results for Au LIII-Edge (a) and Ag K-Edge (c), Detailed with the Contributions from Each Shell (Orange) and Residuals (Purple)^a^

a Summary of the best-fit results for the Au LIII-edge (b) and Ag K-edge (d) on the ANRs-TAT sample.

Analysis of EXAFS experimental data at the Ag K-edge yielded a result similar to the pristine gold nanorod, prior to the functionalization, as previously reported by our group^25^ and was similarly characterized by probing the first shell of coordination for the presence of gold, silver, and bromine as neighbors. Figure 13b,c shows where the best-fit results are compared to the experimental data *k* χ­(*k*), respectively, in *K*[Å-1] and *R*[Å] space. The single scattering paths were obtained from crystallographic models from the Crystallography Open Database^42^ (gold–silver amalgam for Ag–Au, metallic silver for Ag–Ag,^43^ and silver bromide for Ag–Br).^44^ The refinement was achieved by constraining the mean square displacements σ2 of Ag–Br and Ag–Ag to 0.5 and 1.0 × 10^–2^ Å2, respectively, resulting in coordination numbers (C.N.) and distances of 3.4 and 2.88 Å for Ag–Au, 0.7 and 2.68 Å for Ag–Br, and 1.5 and 2.90 Å for Ag–Ag. A detailed outline of the refined parameters is reported in Table 1d. A shell-resolved comparison between experimental data and best-fit results is presented in Table 1c, while a detailed outline of the refined parameters is reported in Table 1d. These results lead to the conclusion that silver is not embedded in the gold lattice (since the sum of the coordination numbers of Ag–Au and Ag–Ag would be higher, closer to 12), but it is instead deposited on the surface of the gold nanorods. Additionally, silver can be either bound with other silver atoms, forming small metallic clusters scattered on the surface, or bound (in the form of Ag­(I)) to bromide atoms from the multilayer of CTAB from above.

## 4 Conclusions

The synthesis of gold nanorods was optimized in terms of size (A.R. = 2.6) and dispersion. This allowed for subsequent functionalization with a modified TAT peptide designed to facilitate delivery to the cell nucleus. Various chemical and physical characterizations of the optimized system were performed, including DLS and Z-potential measurements and UV–vis and FT-IR spectroscopies. Above all, the structural characterizations conducted using synchrotron radiation were crucial for verifying the functionalization and confirming the amazing potential for use in nanomedicine of this engineered system.

## Supplementary Material



## Notes

BE (eV), fwhm (eV), atomic percentages (in the same signal), and proposed assignments for all measured signals and peak position and assignment in the NEXAFS spectra (PDF)
