# Supplementary material for: Peptide-Functionalized Gold Nanorods as a Model to Reach the Cell Nucleus: Synthesis and Structural Characterizations in View of Theragnostic Applications
Source: J Phys Chem B. 2026 Mar 16;130(12):3458–70. doi: 10.1021/acs.jpcb.5c07563 (PMC13034408; doi:10.1021/acs.jpcb.5c07563)
Supplement: Supplementary file 1 [file jp5c07563_si_001.pdf]

Supporting Information

Peptide-functionalized Gold Nanorods as Model to Reach Cell Nucleus: Synthesis and Structural Characterizations in View of Theragnostic Applications

Ludovica Binelli[1,2], Federica Bertelà[1], Simone Amatori[1,3], Diego Lipani[1], Chiara Battocchio[1,2], Giovanna Iucci[1,2], Luca Tortora[1,2], Valentina Dini[4,5], Sveva Grande[4,5], Alessandra Palma[4,5], Marco Ranaldi[1,5], Barbara De Berardis[4,5], Maria G. Ammendolia[4,5], Carlo Mancini-Terraciano[5], Andrea Fabbri [2], Andrea Attili [2], Teresa Scotognella [2,6], Alessandro Giordano[2,6,7], Maria L. Calcagni[2,6,7], Monica Dettin[8], Annj Zamuner[8], Valentin-Adrian Maraloiu[9], Iole Venditti[1,2].*

1 Sciences Department, Roma Tre University, 00146 Rome, Italy;;
2 Istituto Nazionale di Fisica Nucleare (INFN), Sezione di Roma3, Department of Sciences, Roma Tre University, 00146 Rome, Italy
3 CERIC-ERIC, S. S. 14 - km 163,5 in AREA Science Park, Basovizza, Trieste, 34149, Italy
4 National Center for Innovative Technologies in Public Health, Istituto Superiore di Sanità, 00161 Rome, Italy;
5 Istituto Nazionale di Fisica Nucleare (INFN), Sezione di Roma1, Department of Physics, University La Sapienza, 00185 Rome, Italy;
6 Nuclear Medicine Unit, Fondazione Policlinico Universitario A. Gemelli IRCCS, 00168 Rome, Italy;
7 Department of Radiological and Hematological Sciences, Università Cattolica del Sacro Cuore, 00168 Rome, Italy
8 Department of Industrial Engineering, University of Padova, Italy
9 National Institute of Materials Physics, 077125 Magurele, Romania;

* Correspondence: iole.venditti@uniroma3.it;

S1. BE (eV), FWHM (eV), atomic percentages (in the same signal) and proposed assignments for all measured signals.

Table S1. BE (eV), FWHM (eV), atomic percentages (in the same signal) and proposed assignments for all measured signals.

sample	signal	BE (eV)	FWHM (eV)	Atomic %	Assignment
**TAT-x-cys $8*10^{-3}$**	C1s	285.00	1.13	71.5	C-C, C-S
		286.04	1.13	11.2	C-N
		286.99	1.13	5.3	C-O
		288.26	1.13	3.8	NHC=O
		289.32	1.13	5.2	COOH
	$S2p_{3/2}$	161.72	1.45	8.8	S-substrate (TiO_2)
		163.81	1.45	57.8	RS-SR, RSH
		168.37	1.45	33.4	SOx
	N1s	398.50	1.52	12.2	NHCO
		400.16	1.52	82.2	RNH_2
		402.15	1.52	5.6	RNH_3^+
	O1s	530.09	1.76	6.3	TiO_2 substrate
		532.13	1.76	73.4	C=O
		533.69	1.76	19.0	C-O
		535.34	1.76	1.3	Physisorbed H_2O
**AuNRs_TAT-x-cys $8*10^{-3}$**	C1s	285.00	1.34	79.4	C-C, C-S
		286.29	1.34	12.8	C-N
		287.59	1.34	4.6	C-O
		288.77	1.34	2.2	NHC=O
		290.19	1.34	1.0	COOH
	$S2p_{3/2}$ 600eV PE	161.22	1.25	31.8	S-Au sp^3
		163.42	1.25	44.1	RS-SR, RSH
		168.37	1.25	24.1	SOx
	N1s	398.69	1.66	38.0	RNH_2 TAT
		399.97	1.66	62.0	NR_4^+ CTAB
	O1s	530.77	1.94	18.2	C=O
		532.44	1.94	78.1	C-O
		534.11	1.94	3.7	Physisorbed H_2O
	$Au4f_{7/2}$ 600eV PE	83.67	0.59	90.6	Au(0)
		84.39	0.59	9.4	Au(δ^+)
	$Ag3d_{5/2}$	367.57	0.57	91.0	Ag(I)
		368.35	0.57	9.0	Au(0)
	$Au4f_{7/2}$ 260eV PE	83.67	0.57	89.0	Au(0)
		84.35	0.57	11.0	Au(δ^+)
	$S2p_{3/2}$ 260eV PE	161.28	1.22	24.5	S-Au sp^3
		163.46	1.22	52.2	RS-SR, RSH
		168.32	1.22	23.3	SOx
	$Br3d_{5/2}$ 260eV PE	69.08	1.22	86.0	CTAB
		69.16	1.22	14.0	$Ag^+/Br^-/CTA^+$ complex

S2. Peak position and assignment in the NEXAFS spectra

Table S2; peak position and assignment in the NEXAFS spectra			
		Photon Energy (eV)	
edge	assignment	cys-TAT	AuNRs-cys-TAT
C K	$\pi^*_{C=C}$	285.4	285.5
	$\pi^*_{C=O}$	288.7	288.7
	σ^*	295.5	295.9
	σ^*	302	302
N K	π^*_{arg}	399.3<br>400.3	399
	π^*_{pept}	402.0	402.1
	σ^*_{N-H}	408.2	408
	σ^*_{N-C}	413	411
O K	$\pi^*_{C=O}$	532.5	532.6<br>533.8
	σ^*	540.8	540
